# Hematological profile, inflammatory markers and serum liver enzymes in COVID 19 positive children vs. COVID 19 negative ones—a comparative study

**DOI:** 10.3389/fped.2024.1334591

**Published:** 2024-02-15

**Authors:** Mirela Luminița Pavelescu, Alexandru Dinulescu, Alexandru-Sorin Păsărică, Irina Dijmărescu, Daniela Păcurar

**Affiliations:** ^1^ Departament of Pediatrics, “Carol Davila” University of Medicine and Pharmacy, Bucharest, Romania; ^2^ Department of Pediatrics, “Grigore Alexandrescu” Emergency Children's Hospital, Bucharest, Romania

**Keywords:** SARS CoV-2, COVID-19, complete blood count, C-reactive protein, transaminases

## Abstract

**Background:**

Complete blood count, C-reactive protein and transaminases are routine laboratory parameters investigated in children with infections, including COVID 19. We aimed to evaluate the diagnostic accuracy of these parameters in children diagnosed with COVID 19.

**Methods:**

At the time of admission, children with COVID 19 suggestive symptoms were tested RT-PCR for SARS CoV-2 and were allocated to either the study group (RT-PCR SARS CoV-2 positive) or control group (RT-PCR SARS CoV-2 negative). All children were evaluated by complete blood count, CRP, and transaminases.

**Results:**

When comparing the two groups, we identified significantly lower values for leukocytes (*p* < 0.001), neutrophils (*p* < 0.001), lymphocytes (*p* < 0.001) and thrombocytes (*p* = 0.014), but no significantly different values for CRP (*p* = 0.916) and monocytes (*p* = 0.082). A diagnostic score for COVID-19 was compiled using the abovementioned parameters—presence of fever, number of lymphocytes and aspartate-aminotransferase. Performance was tested, showing a positive discrimination value (AUC of 0.703)—81.5% sensitivity, 50.6% specificity.

**Conclusions:**

The leukocytes, neutrophils and lymphocytes have significantly lower values in COVID-19 children. The proposed score based on the presence of fever the values of lymphocytes and AST has a good sensitivity in predicting COVID-19 infection.

## 1 Introduction

COVID-19 disease was first described in 2019 in Wuhan, China and it later spread throughout the world, becoming a pandemic (1). World Health Organization (WHO) initially named the virus the “novel coronavirus 2019”, being subsequently renamed SARS-CoV-2, an acronym from “severe acute respiratory syndrome coronavirus 2”. The disease was called COVID-19, an acronym from “coronavirus disease 2019” (2). The clinical picture of this viral infection is variable, from asymptomatic to acute respiratory failure (3). In the first stages of the pandemic, the incidence in the pediatric population was low, children accounting for 1%–2% of the reported cases, mostly non-severe (4). However, with the emergence of the delta variant, COVID-19 pediatric patients were on the rises (5).

Coronaviruses are enveloped RNA viruses that infect a variety of species, including humans. They cause respiratory diseases, varying from mild cases, like common cold, to severe ones, like acute respiratory failure. The coronaviruses that infect humans are classified as low pathogenic—hCOVs and highly pathogenic ones—Middle East respiratory syndrome coronavirus (MERS-CoV) and severe acute respiratory syndrome coronavirus (SARS-CoV). The new Coronavirus was classified as a severe acute respiratory syndrome coronavirus by the International Committee on Taxonomy of Viruses (ICTV) and later named SARS-CoV 2 (4, 6).

WHO and Center for Disease Control (CDC) established the case definition for coronavirus disease 2019 (COVID-19) suspected and probable cases, based on epidemiological criteria, clinical, laboratory and radiological data (7–9).

The symptoms of COVID-19 include fever, cough, dyspnea, dysphagia, cephalalgia, fatigue and myalgia, making it difficult to discriminate this specific disease from other respiratory ailments (10–13). The gold-standard test for the diagnosis of COVID-19 is quantitative fluorescence-real time reverse transcriptase polymerase chain reaction (RT-PCR). The accuracy of the test varies with the quality of the sample, the stage of disease and the viral clearance or multiplication in that moment. Its sensitivity is estimated between 70% and 98%, with a specificity of approximately 95% (14–16).

SARS CoV-2 infection may be associated with alteration of the white blood cell count (WBC). In adults, leukopenia is identified in approximately 25% of the patients with COVID-19, most of them having lymphocytopenia (17). Lymphocytopenia is more frequent in severe cases when compared to mild ones (18). High neutrophils were also reported in COVID-19, and the number of neutrophils was also found to be increased in the nasopharyngeal epithelium and in the lungs (19). In children, low leukocytes and lymphocytopenia are rare and may correlate with the severity of the infection (20).

The platelet count may be low in COVID-19, but the decrease is generally mild (100–150 × 109/L) and the incidence varies with the severity of the diseases. Severe thrombocytopenia was rarely reported (21).

In COVID-19, the liver may be injured directly or indirectly by the virus, through different mechanisms -binding ACE-2 receptors expressed on the cholangiocytes, cytokine dysregulation, microangiopathy, hypoxia, shock or sepsis, hepatotoxicity of the antiviral drugs (22, 23).

Thus, liver enzymes may be used as additional markers for the severity of COVID-19 (24–26). It is uncertain whether this is applicable in the pediatric population, but elevated transaminases may be associated with the severity of the disease (27–29).

The present research aimed to evaluate the impact of the leukocyte count, thrombocytes, C-reactive protein, and transaminases on the diagnosis of COVID-19 in children.

We propose a prediction score aimed to raise suspicion of COVID-19 in children evaluated in primary care facilities, where specific testing might not be readily available. The score includes clinical symptoms and basic laboratory tests, accessible in most health stations.

## 2 Methods

We conducted a retrospective and prospective study that included patients hospitalized between March 2020 and January 2022 in the Pediatrics Department of “Grigore Alexandrescu” Emergency Children's Hospital in Bucharest, Romania.

Inclusion criteria: Patients aged 0–17 years old with COVID-19 suggestive symptoms, based on the CDC, WHO and national COVID-19 case definition: fever or chills, cough, dyspnea, fatigue, myalgia, headache, anosmia, ageusia, sore throat, nasal congestion or rhinorrhea, nausea or vomiting and diarrhea.

Exclusion criteria: Children with incomplete laboratory data. Personal or familial history of hematological, liver, neurological or congenital diseases.

All patients were tested for SARS CoV-2 infection by RT-PCR. Based on the results, patients were distributed in the corresponding group: study group—subjects with positive RT-PCR for SARS CoV-2 and control group—subjects with negative RT-PCR for SARS CoV-2.

Information regarding the clinical symptoms was noted, together with laboratory data—number of leukocytes, neutrophils, lymphocytes and thrombocytes, value of C-reactive protein (CRP) and transaminases. Laboratory data was compared between the two established groups. A diagnostic score based on clinical and laboratory features was compiled and was subsequently tested for performance.

All data was organized and illustrated using Microsoft Office Excel/Word 2013 and was analyzed using IBM SPSS Statistics 25. Quantitative variables were tested for normal distribution using the Kolmogorov–Smirnov test and were written as medians with interquartile ranges (IQR). Qualitative variables were tested between independent groups using Mann–Whitney *U* tests and correlations between them were made using Spearman's rho coefficients. Receiver operating characteristic (ROC) curves were used for establishing cut-off values in prediction of COVID-19 infection. Logistic regression models were verified for goodness-of-fit and used for estimating the prediction value of laboratory parameters in case of COVID-19 infection (in univariate and multivariate models) and the quality of prediction was illustrated using ROC curves. The performance of the score was evaluated based on the ROC curve, which was used to assess positive and negative predictive values, defining its discrimination utility, determined by the area under the curve (AUC).

## 3 Results

### 3.1 Demographics

Out of 857 potentially eligible patients, 664 were selected based on the inclusion and exclusion criteria—a number of 116 subjects were excluded due to lack of data or presence of comorbidities. The demographic characteristics of the study group are presented in Table 1. The median age for the study group was 6 months, IQR 2–22 months. Regarding the gender, 368 (55.42%) of the children were male and 296 (44.58%) females.

**Table 1:** Demographic characteristics of the patients included in the study.

	Number (%)	Gender (%)		Median age—months (IQR)
		Male	Female	
COVID-19 positive patients	348 (52.4%)	185 (53.16%)	163 (46.84%)	6 (2–19)
COVID-19 negative patients	316 (47.6%)	183 (57.91%)	133 (42.09%)	7.5 (2–28)

### 3.2 Clinical findings

The most common symptom we noted was fever (90.8%). In order of frequency, others clinical findings were rhinorrhea (64.94%), coughing (56.32%) and dyspnea (9.79%). Clinical findings are presented in Table 2.

**Table 2:** Clinical findings in COVID-19 patients included in the study, in order of frequency.

Clinical symptoms/signs	Number (%)
Fever	316 (90.8%)
Rhinorrhea	226 (64.94%
Coughing	196 (56.32%)
Vomiting	65 (18.67%)
Diarrhea	60 (17.24%)
Dyspnea	34 (9.79%)

### 3.3 Laboratory parameters

None of the laboratory parameters followed a normal distribution according to the Kolmogorov–Smirnov test (*p* < 0.05).

There were no significant differences for age (*p* = 0.345), CRP (*p* = 0.916) and monocytes (*p* = 0.082) between the two groups. Significantly lower values were found for the leukocyte count (*p* < 0.001), neutrophil count (*p* < 0.001), lymphocyte count (*p* < 0.001) and thrombocyte count (*p* = 0.014) in the COVID-19 positive group. Significantly higher values were found in the COVID-19 positive group for transaminases—alanine-aminotransferase (ALT) (*p* = 0.001) and aspartate-aminotransferase (AST) (*p* < 0.001). Median values for age and laboratory parameters in the respective groups are presented in Table 3.

**Table 3:** Median comparison for age and laboratory parameters between the two groups.

	COVID-19 positive (*N* = 348)	COVID-19 negative (*N* = 316)	*p* value
	Median (IQR)	Median (IQR)	
Age (months)	6 (2–19)	7.5 (2–28)	0.345
CRP (mg/dl)	0.3 (0.11–0.77)	0.31 (0.09–0.95)	0.916
Leukocytes ×10^9^/L	6,95 (5,03–9,8)	9,63 (6.77–13.1)	**<0.001**
Neutrophils ×10^9^/L	2.61 (1.59–4.41)	3.62 (2.14–6.7)	**<0.001**
Lymphocytes ×10^9^/L	2.47 (1.26–4.51)	3.7 (2–6)	**<0.001**
Monocytes ×10^9^/L	0.8 (0.52–1.17)	0.86 (0.51–1.3)	0.082
Thrombocytes ×10^9^/L	320.5 (260.25–411.25)	354 (259.5–444.5)	**0.014**
ALT (U/L)	25 (17.25–32)	21 (16–29)	**0.001**
AST (U/L)	46 (37–59)	41 (32–53.75)	**<0.001**

The bolded values are statistically significant results in the tests we used.

### 3.4 Logistic regression models

We represented logistic regression models for COVID-19 infections (Table 3). In univariate models, neutrophils (*p* < 0.001), lymphocytes (*p* < 0.001), thrombocytes (*p* = 0.006) and AST (*p* = 0.005) have a significant prediction on COVID-19 infection, as such:

- Each increase of 10^3^ units of neutrophils decrease the odds of having COVID-19 infection by 1.136 times (95% CI: 1.08–1.196);
- Each increase of 10^3^ units of lymphocytes decrease the odds of having COVID-19 infection by 1.18 times (95% CI: 1.108–1.254);
- Each increase of 10^3^ units of thrombocytes decrease the odds of having COVID-19 infection by 1.002 times (95% CI: 1–1.003);
- Each increase of 1 unit of AST increases the odds of having COVID-19 infection by 1.010 times (95% CI: 1.003–1.018).

A multivariate regression model was constructed using neutrophils, lymphocytes, thrombocytes, and AST as prediction variables. In this model, only neutrophils (*p* < 0.001), lymphocytes (*p* < 0.001) and AST (*p* = 0.013) have a significant prediction on COVID-19 infection, as such:

- Each increase of 10^3^ units of neutrophils decrease the odds of having COVID-19 infection by 1.141 times (95% CI: 1.082–1.204);
- Each increase of 10^3^ units of lymphocytes decrease the odds of having COVID-19 infection by 1.207 times (95% CI: 1.124–1.297);
- Each increase of 1 unit of TGO increases the odds of having COVID-19 infection by 1.009 times (95% CI: 1.002–1.017).

In Table 4 we have represented the univariable and multivariable logistic regression analysis of the laboratory parameters for COVID-19 infection.

**Table 4:** Logistic regression for COVID-19 infection.

Variable	Univariable		Multivariable	
	OR (95% CI)	*p*	OR (95% CI)	*p*
Age	0.825 (0.607–1.121)	0.219	—	—
Gender	0.834 (0.609–1.142)	0.258	—	—
Leukocytes	1.000 (1.000–1.000)	0.537	—	—
Neutrophils	**0.880** (**0.836–0.926)**	**<0**.**001**	**0.876 (0.830–0.924)**	**<0.001**
Lymphocytes	**0.847** (**0.797–0.902)**	**<0**.**001**	**0.828 (0.771–0.889)**	**<0.001**
Thrombocytes	**0.998** (**0.997–1.000)**	**0**.**006**	1.000 (0.999–1.001)	0.991
AST	**1.010** (**1.003–1.018)**	**0**.**005**	**1.009 (1.002–1.017)**	**0.013**
ALT	1.009 (0.999–1.019)	0.070	—	—

The bolded values are statistically significant results in the tests we used.

### 3.5 Correlation between laboratory parameters

Using the Spearman correlation analysis, neutrophils have shown a statistically significant but weak correlation with AST and ALT (*p* < 0.001, *R* = 0.289, R = 0.225). Lymphocytes correlate moderately with thrombocytes (*p* < 0.001, *R* = 0.429) and weakly with AST (*p* = 0.001, *R* = 0.135). Thrombocytes have a very weak correlation with the AST (*p* < 0.001, *R* = 0.193) and there is a moderate correlation between the transaminases (*p* < 0.001, *R* = 0.599). The results are presented in Table 5.

**Table 5:** Correlations between laboratory parameters.

Parameter	Neutrophils	Lymphocytes	Thrombocytes	AST	ALT
Neutrophils	—	0.060, *R*** **=** **−0.073	0.373 *R*** **=** **0.035	**<0.001,** ***R* = −0.289**	**<0.001,** ***R* = −0.225**
Lymphocytes	0.060, *R*** **=** **−0.073	—	**<0.001,** ***R* = 0.429**	**0.001,** *R*** **=** **0.135	0.051, *R*** **=** **0.076
Thrombocytes	0.373 *R*** **=** **0.035	**<0.001**, ***R* = 0.429**	—	**<0.001,** *R*** **=** **0.193	0.911, *R*** **=** **0.004
AST	**<0.001,** ***R* = −0.289**	**0.001,** ***R* = 0.135**	**<0.001,** ***R* = 0.193**	—	**<0.001,** ***R* = 0.599**
ALT	<0.001, *R*** **=** **−0.225	0.051, *R*** **=** **0.076	0.911, *R*** **=** **0.004	**<0.001,** ***R* = 0.599**	—

The bolded values are statistically significant results in the tests we used.

### 3.6 The ROC curve of laboratory parameters

**Figure 1:**
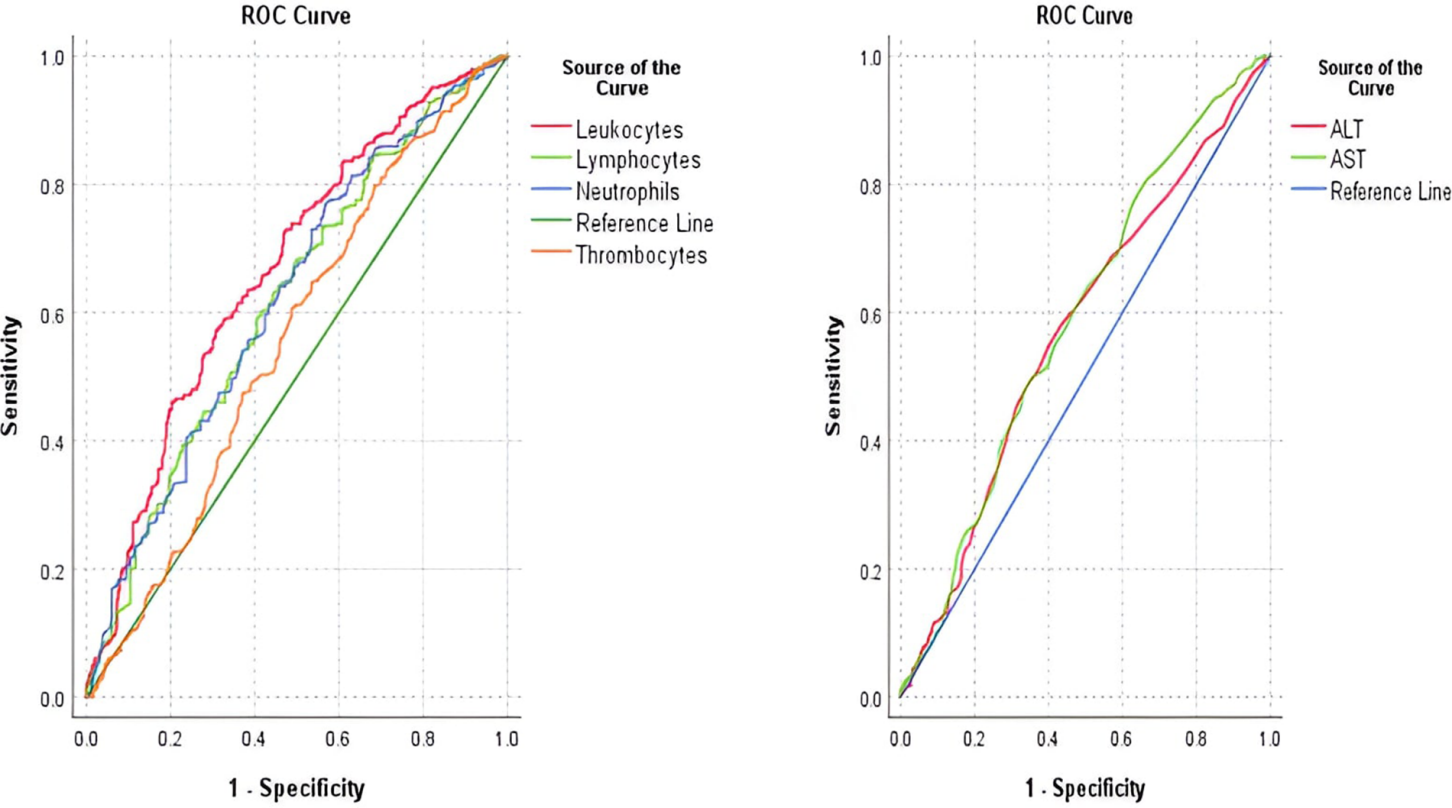
AUC-ROC curve for leukocytes, lymphocytes, neutrophils, and thrombocytes in predicting COVID-19 positive cases (left); AUC-ROC curve for AST and ALT in predicting COVID-19 positive cases.

When applying the ROC curve, identified cut-off values for each of the laboratory parameters showed poor discrimination strength (AUC < 0.7). Sensitivity ranged from 49.7% to 76.7%, while specificity ranged from 43.4% to 69.3%. The ROC curve and the AUC-ROC analysis of parameters are presented in Figure 1 and Table 6.

**Table 6:** AUC-ROC analysis of the laboratory parameters in predicting positive COVID-19 cases.

Variable	Cut-off	Youden index	AUC	95% CI	*p*	Sensitivity	Specificity
Leukocytes	7.475	0.265	0.666	0.625–0.707	**<0** **.** **001**	57.2%	69.3%
Neutrophils	4.595	0.201	0.622	0.579–0.664	**<0** **.** **001**	76.7%	43.4%
Lymphocytes	3.065	0.187	0.618	0.575–0.660	**<0** **.** **001**	59.2%	59.5%
Thrombocytes	351.5	0.119	0.555	0.511–0.599	**0** **.** **014**	60.6%	51.3%
AST	23.5	0.147	0.572	0.528–0.616	**0** **.** **001**	54.6%	60.1%
ALT	46.5	0.146	0.589	0.546–0.633	**<0** **.** **001**	49.7%	64.9%

The bolded values are statistically significant results in the tests we used.

**Figure 2:**
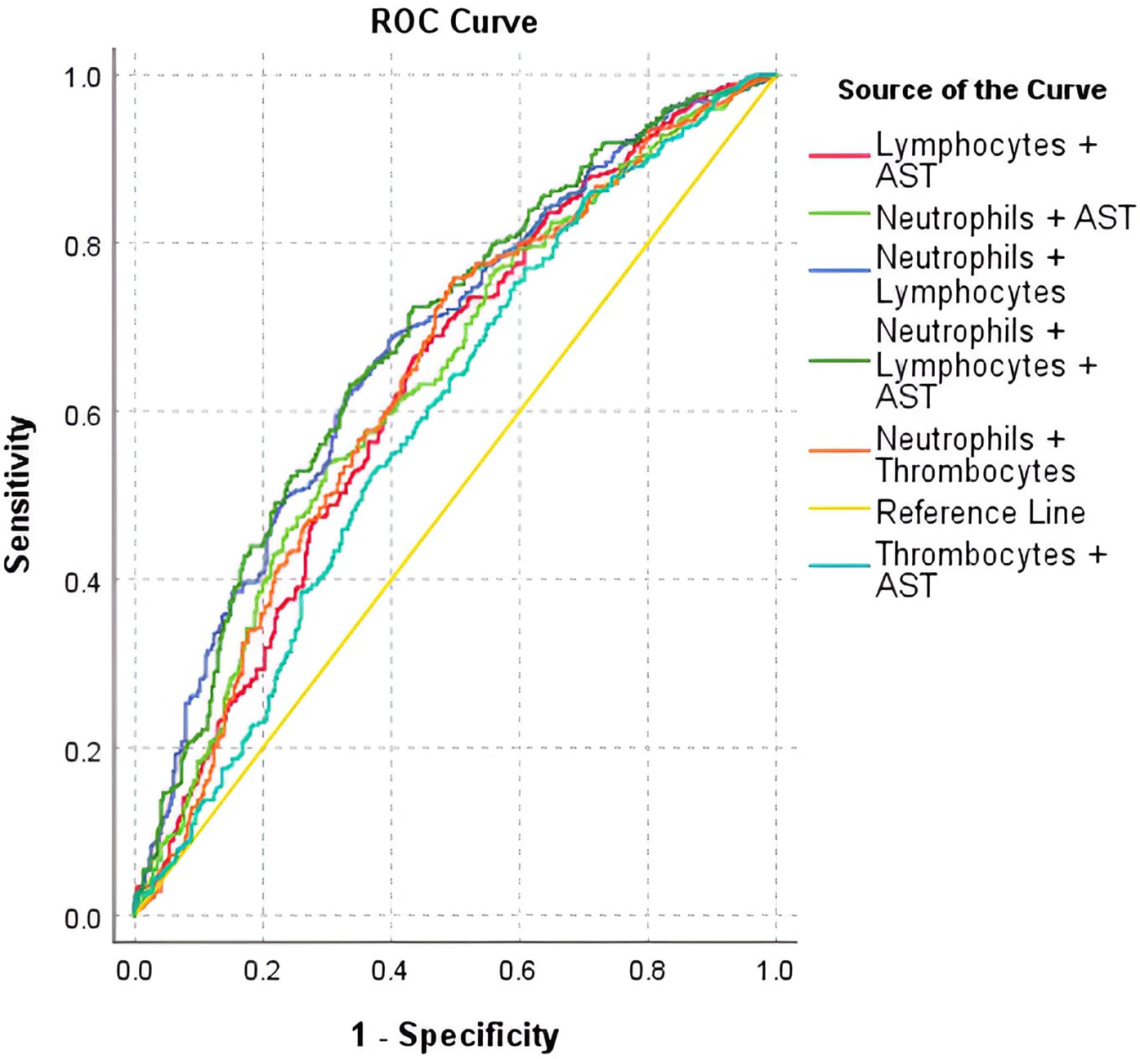
AUC-ROC curve for combined laboratory parameters in predicting positive COVID-19 cases.

When using the ROC curve for combined laboratory parameters, the discrimination strengths remained poor (AUC < 0.7), sensitivity ranging from 53.7% to 77% and specificity from 39.2% to 70.3%. The ROC curve and the AUC-ROC analysis of combined parameters are presented in Figure 2 and Table 7.

**Table 7:** AUC-ROC analysis of combined laboratory parameters in predicting positive COVID-19 cases.

Variable	Youden index	AUC	95% CI	*P*	Sensitivity	Specificity
Neutrophils & Lymphocytes	0.288	0.678	0.638–0.719	**<0** **.** **001**	62.1%	66.8%
Neutrophil & Thrombocytes	0.262	0.638	0.595–0.680	**<0** **.** **001**	75.9%	50.3%
Neutrophils & AST	0.240	0.636	0.594–0.679	**<0** **.** **001**	53.7%	70.3%
Lymphocytes & AST	0.230	0.634	0.591–0.676	**<0** **.** **001**	66.4%	56.6%
Thrombocytes & AST	0.163	0.594	0.551–0.637	**<0** **.** **001**	77%	39.2%
Neutrophils & Lymphocytes & AST	0.297	0.684	0.644–0.725	**<0** **.** **001**	63.2%	66.5%

The bolded values are statistically significant results in the tests we used.

### 3.7 Proposed prediction score

**Figure 3:**
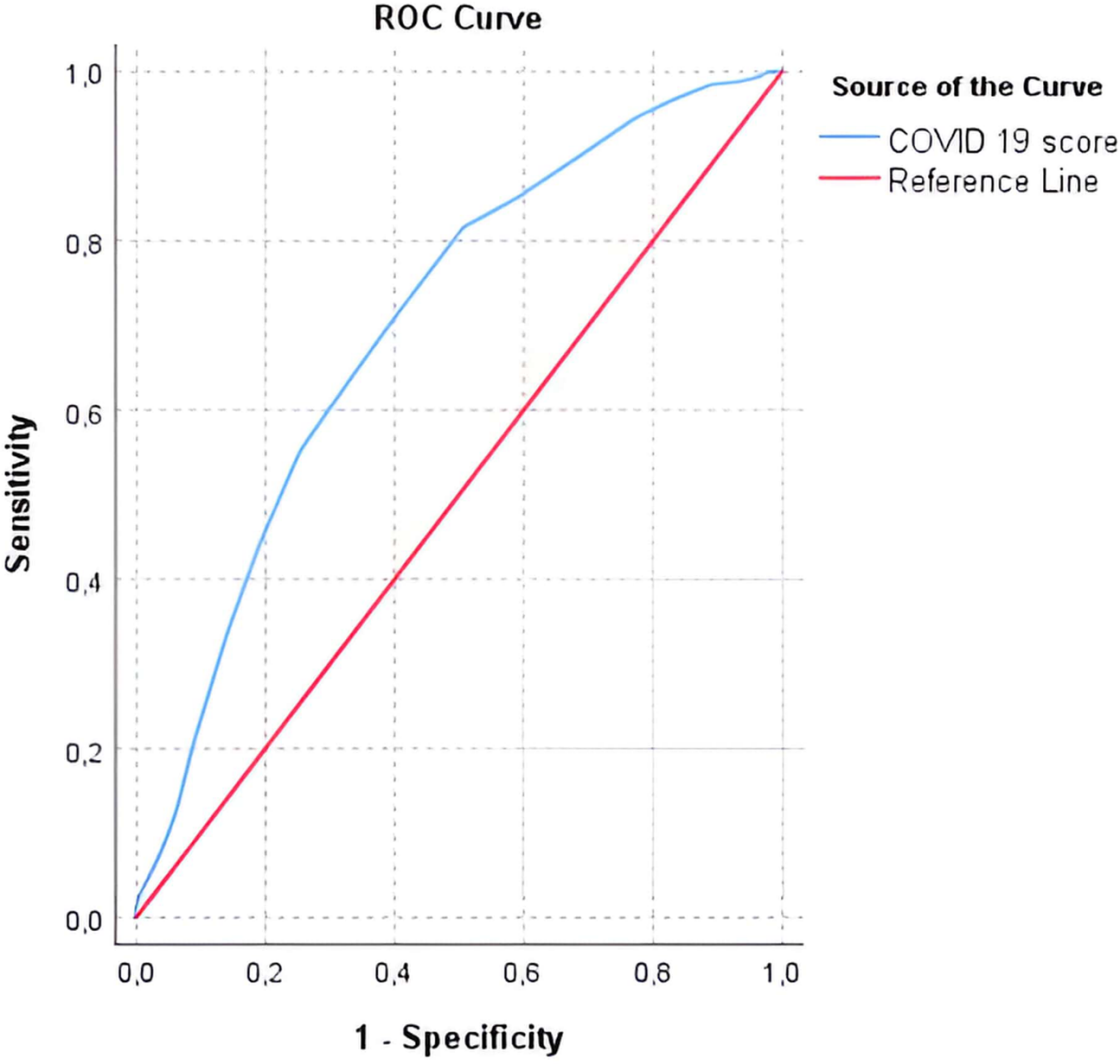
AUC-ROC curve for COVID-19 prediction score.

A prediction score was elaborated, taking into account the presence of fever and laboratory parameters (neutrophils, lymphocytes, AST). The possible value of the score ranges from −13 points to +13 points. When applying the ROC curve, for a cut-off value of −0.5 points, the sensitivity was 81.6% and the specificity 50.6%, with an acceptable discrimination power (AUC = 0.703). The score is presented in Table 8 and the AUC-ROC curve is presented in Figure 3. Table 9 presents the AUC-ROC for COVID 19 score in predicting COVID-19 positive cases.

**Table 8:** Prediction score for COVID-19.

Neutrophils (×10^9^/L)		Lymphocytes (×10^9^/L)		AST (U/L)	
<1	+4	<2	+4	>100	+4
1–2	+3	2–3	+3	90–100	+3
2–3	+2	3–4	+2	80–90	+2
3–4	+1	4–5	+1	70–80	+1
4–5	0	5–6	0	60–70	0
5–6	−1	6–7	−1	50–60	−1
6–7	−2	7–8	−2	40–50	−2
7–8	−3	8–9	−3	30–40	−3
>8	−4	>9	−4	<30	−4

Fever—present +1/absent −1.

**Table 9:** AUC-ROC for COVID 19 score in predicting COVID-19 positive cases.

Variable	Cut-off	Youden index	AUC	95% CI	*p*	Sensitivity	Specificity
COVID 19 prediction score	−0.5	0.32	**0.703**	0.664–0.743	**<0.001**	81.6%	50.6%

The bolded values are statistically significant results in the tests we used.

## 4 Discussion

The current study included 664 pediatric patients aged 0–17 years old, evaluated for symptoms that fit the COVID-19 case definition, according to guidelines from CDC, WHO and local authorities. Our research reports significantly lower values for leukocytes, neutrophils and lymphocytes in the COVID-19 positive group when compared with negative one, observation similar to findings in adult populations.

Most of the COVID-19 studies that analyzed pediatric subjects describe fever and coughing as main symptoms (41.5%–62.4%, respectively 40.6%–55.9% of cases). In our research, we also report these symptoms as most common in the study group. Coughing was identified in the current study in similar percentages than in other papers (56.32%), but we report a higher presence of fever when compared to the literature (90.8%). The high incidence of fever in our study group can be explained by the fact that our data was retrieved from patients admitted to the hospital (30–33).

Ferrari et al. compared two adult groups with suggestive symptoms for COVID-19, 105 positive patients and 102 negative ones, concluding that leukocytes, neutrophils, lymphocytes and monocytes had statistically significant lower values in the COVID-19 positive group (*p* < 0.005), supporting our results. Even though our study included a higher number of patients, we did not find a statistically significant decrease of the monocytes. The abovementioned research also reported a significantly higher value for CRP in the COVID-19 positive group, unlike our research. No significant difference was reported for the platelets’ values, in contradiction with our reports (34). Another research, conducted by Huang et al. in 2020, which included 41 adult COVID-19 patients, 13 of them admitted in the Intensive Care Unit (ICU), reported notably higher leukocytes and neutrophils and lower lymphocytes in ICU patients when compared to non-ICU ones. There was no statistically significant difference reported for hemoglobin and platelets (35). Cheng et al. analyzed 33 patients with pneumonia, 11 COVID-19 positive and 22 non-COVID-19, reporting that the COVID-19 group had lower leukocytes and lower platelets, but higher CRP (36). Dobrijević et al. published a study that included 232 children with COVID-19 in which they concluded that the number of platelets was not significant in predicting the disease (37).

The discrepancies identified among our research and the abovementioned studies may be justified not only by the age difference of the study groups, but also the different number of patients analyzed. Hoang et al. conducted a systematic review that included 7,780 children with COVID-19, finding a mildly decrease of neutrophils (44.4%) and a marginally elevation of lymphocytes (39.9%). The decrease of neutrophils is consistent with the results of the current research, but the increase of lymphocyes is in contradiction with our conclusions (30).

Sava et al. analyzed a group of 234 COVID-19 pediatric cases, describing leukopenia (20.4%), lymphopenia (35%) and neutropenia (17.5%) as the most common changes in WBC. Their data supports our results (38).

Moreover, a study published by Alkan et al, found lymphopenia to be the most common hematological shift, reporting it in 36% of cases. This research included 663 COVID-19 pediatric patients (39).

Another research conducted by Kaftan et al. in 2021 noted a significant increase of the CRP in COVID-19 patients when compared to negative ones. The research included 566 adult subjects that had had contact with a COVID-19 patient or had suggestive symptoms such as cough, anosmia, ageusia, myalgia, fever, dyspnea, or dysphagia. The subjects were divided in two groups based on the RT-PCR test—361 of them were COVID-19 positive, 205 negatives. The median value for CRP in the positive group was statistically significant higher 18 (46) mg/L than in the negative one 6 (9) mg/dl (*p* < 0.001) (40).

According to Alqahtani et al, significant higher values were identified for ALT (25 U/L vs. 21 U/L, *p* = 0.001) and AST (46 U/L vs. 41 U/L, *p* < 0.001) in COVID-19 positive children when compared to negative ones (26).

The prevalence of ALT elevations among adult patients with COVID-19 ranged from 4% to 39% in different studies. The prevalence of AST elevations ranged from 4% to 58%. The prognostic value of abnormal liver functional tests in COVID-19 is unclear (41).

Liver enzymes in pediatric patients with COVID-19, based on published case series, were found to be mildly elevated, with an overall prevalence of approximately 29% (27).

A study published in 2022 which included 3,380 adults with COVID-19 found high ALT at admission in 2,698 of the patients (70.4%). Higher values were also found for AST (44.4%), alkaline phosphatase (16.1%) and total bilirubin (5.9%). These biochemical alterations were more commonly associated with disease severity (42).

A meta-analysis conducted by Qi et al, that included 37 studies based on pediatric patients with SARS-CoV-2 infection, described most common findings as following: leukopenia in 27 studies, lymphopenia in 25 studies, elevation of the CRP in 24 studies and elevated values of the AST in 19 studies (32).

A review published by Bitar et al. in 2023 reported elevatation of transminases in children with COVID-19, 20%–50% rising in AST and almost 35% in ALT (43).

In logistic regression models—univariate models for COVID-19 infections, neutrophils (*p* < 0.001), lymphocytes (*p* < 0.001), thrombocytes (*p* = 0.006) and AST (*p* = 0.005) have shown a significant prediction value for COVID-19 infection in pediatric patients. The results of the multivariable logistic regression showed that neutrophils (*p* < 0.001), lymphocytes (*p* < 0.001), thrombocytes (*p* = 0.006) and AST (*p* = 0.005) values are associated with the risk of COVID-19 infection.

In our study, from the Spearman correlation analysis we concluded that lymphocytes, thrombocytes, ALT, and AST had a moderate positive correlation with COVID-19 (*p* < 0.05; *r* = 0.4–0.6). The ROC curve illustrated a low sensitivity and specificity for discriminating COVID 19 in pediatric patients by using leukocytes, neutrophils, lymphocytes, thrombocytes, AST and ALT.

Based on our data and the findings in the literature we compiled a score that uses the presence of fever and laboratory parameters (neutrophils, lymphocytes and AST), for which a cut-off value of −0.5 points provides 81.6% sensitivity and 50.6% specificity, but with an acceptable discrimination (AUC = 0.703).

### 4.2 Limitation of the study

The severity of the disease was not defined in relevance to the changes of the laboratory parameters. Some laboratory markers such as procalcitonin, LDH, ferritin and D dimers were not included, because we did not intend to evaluate the severity.

## 5 Conclusions

In COVID 19 positive pediatric patients the WBC, neutrophils, lymphocytes, and thrombocytes have significantly lower values than in negative children.

The values of the liver enzymes, especially AST, are significantly higher in pediatric patients with COVID 19. Still, their values are only mildly moderate increased.

The combination of fever, lymphocytes and AST in a score system has an accepted sensitivity in predicting COVID-19 infection in pediatric patients that fit the COVID 19 case definition.

Because nowadays COVID-19 is considered a common disease, even though antigen testing is widespread, regular laboratory tests such as complete blood count and liver enzymes, when used together, may raise awareness of potential cases.

## Data Availability

The raw data supporting the conclusions of this article will be made available by the authors, without undue reservation.
