# Corrigendum: Hematological profile, inflammatory markers and serum liver enzymes in COVID 19 positive children vs. COVID 19 negative ones—a comparative study

**DOI:** 10.3389/fped.2024.1401564

**Published:** 2024-04-03

**Authors:** Mirela Luminița Pavelescu, Alexandru Dinulescu, Alexandru-Sorin Păsărică, Irina Dijmărescu, Daniela Păcurar

**Affiliations:** ^1^ Department of Pediatrics, “Carol Davila” University of Medicine and Pharmacy, Bucharest, Romania; ^2^ Department of Pediatrics, “Grigore Alexandrescu” Emergency Children's Hospital, Bucharest, Romania

**Keywords:** SARS CoV-2, COVID-19, complete blood count, C-reactive protein, transaminases

A Corrigendum on Hematological profile, inflammatory markers and serum liver enzymes in COVID 19 positive children vs. COVID 19 negative ones—a comparative study Pavelescu ML, Dinulescu A, Păsărică A-S, Dijmărescu I and Păcurar D (2024) Front. Pediatr. 12:1334591. doi: 10.3389/fped.2024.1334591

In the published article the Acknowledgments statement was not included. The Acknowledgments statement appears below.

